# Innovative Thoughts on Treating Diabetes from the Perspective of Traditional Chinese Medicine

**DOI:** 10.1155/2015/905432

**Published:** 2015-10-04

**Authors:** Bing Pang, Qiang Zhou, Tian-Yu Zhao, Li-Sha He, Jing Guo, Hong-Dong Chen, Lin-Hua Zhao, Xiao-Lin Tong

**Affiliations:** ^1^Department of Endocrinology, Guang'anmen Hospital of China Academy of Chinese Medical Sciences, Beijing 100054, China; ^2^Department of Digestion, Beijing Hospital of Traditional Chinese Medicine, Capital University of Medicine Sciences, Beijing 100010, China; ^3^Laboratory of Molecular Biology, Guang'anmen Hospital of China Academy of Chinese Medical Sciences, Beijing 100054, China

## Abstract

The rapidly increasing incidence of diabetes mellitus (DM) is becoming a major public health issue. As one of the important parts in complementary and alternative therapies, traditional Chinese medicine (TCM) is promising in treating DM. In this review, we summarize new thoughts on treating DM that aim to improve the clinical efficacy of TCM from the perspectives of principle, methods, formula, herbs, and doses. Our approach is as follows: principle: we use a combination of symptoms, syndromes, and diseases as a new mode for treating diabetes; methods: emphasizing heat-clearing in the early and middle stage of T2DM and invigorating blood circulation throughout the whole process of T2DM are two innovative methods to treat T2DM; formulas and herbs: choosing formulas and herbs based on the combination of TCM theory and current medicine. We will emphasize four strategies to help doctors choose formulas and herbs, including treatment based on syndrome differentiation, choosing herbs of bitter and sour flavors to counteract sweet flavor, choosing formulas and herbs aimed at main symptoms, and using modern pharmacological achievements in clinical practice; dose: reasonable drug dose plays an important role in the treatment of DM and a close relationship exists between dose and clinical efficacy.

## 1. Introduction

**Figure 1:**
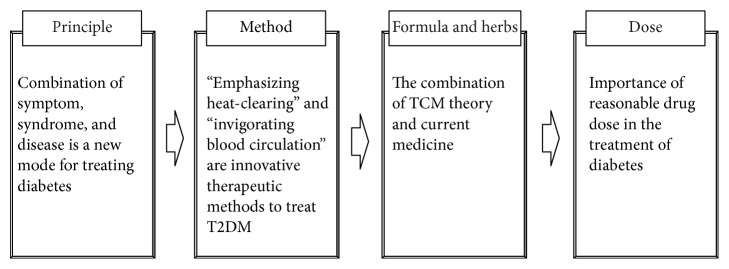
The scheme figure of the innovative thoughts in the treatment of diabetes.

Diabetes mellitus (DM) is a chronic metabolic disorder caused by either absolute deficiency in insulin secretion or reduction in the biological effectiveness of insulin. The global prevalence of DM among adults aged 20–79 years was 8.3% in 2013 [1]. As one of the largest developing countries, China has the biggest population of patients with DM with 92.4 million, which account for 9.7% of the adult population. In addition, 148.2 million adults (15.5%) have prediabetes [2]. DM has a significant impact on the quality of life and life expectancy of people as well as on the economic burden on the health care system. Therefore, it represents a major public health issue [3]. Type 2 diabetes mellitus (T2DM) is the predominant form of DM and accounts for 90–95% of the diabetic populations, due to an increased number of elderly patients and a greater prevalence of obesity and sedentary lifestyles [4, 5]. Management of T2DM is still a challenge and the standard therapy for T2DM includes balanced diet, appropriate exercise, use of oral hypoglycemic drugs, and/or subcutaneous insulin injections [6]. Although considerable progress has been made regarding hypoglycemic drugs and insulin, Western medicine still has some limitations. Traditional Chinese medicine (TCM) has a long history of more than 2000 years in treating DM [7, 8], and there are several advantages in treating DM with TCM, including lower rate of toxicity and/or side effects, holistic regulation of metabolic problems, reversal of risk factors leading to T2DM, and delaying diabetic complications. Due to the differences in etiology, pathogenesis, diagnosis, and interventions between traditional Xiaoke disease and T2DM, several new therapeutic thoughts have been recently proposed. In this review, we summarized these thoughts based on principle, method, formula, herbs, and dose through literature analysis in both English and Chinese search engines to guide clinicians in treating T2DM. The scheme figure of the innovative thoughts in the treatment of diabetes is shown in Figure 1.

## 2. Principle: Combination of Symptom, Syndrome, and Disease Is a New Mode for Treating Diabetes

**Figure 2:**
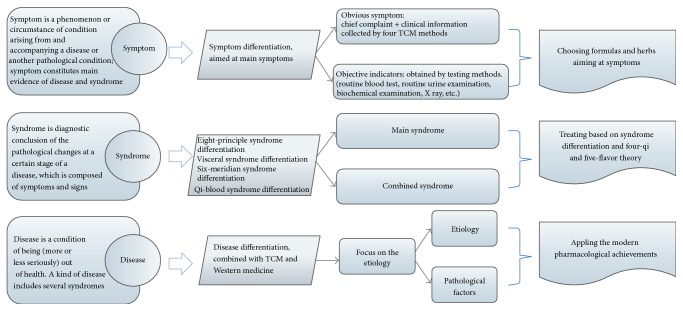
The mode of combining symptom, syndrome, and disease.

The “combination of symptoms, syndrome, and disease” has been widely used in the treatment of several chronic difficult diseases [9–11]. The mode of combining symptoms, syndromes, and diseases is shown in Figure 2. Syndromes, also known as “zheng” or “pattern,” are the abstraction and generalization of the pathological changes at a certain stage of a disease, which shows the essence of a disease more deeply and completely [12]. Syndrome differentiation is diagnosed through comprehensive consideration of symptoms and signs (tongue appearance and pulse feeling included) and has implications for determining the cause, location, and nature of the disease and the patient's physical condition, as well as the trend of development [13]. As an example for syndrome differentiation, one T2DM patient with obesity, reddened complexion, stuffiness and fullness in the abdomen, red tongue, yellow-greasy coating, and slippery pulse may suffer from typical phlegm and heat stasis syndrome, while the other T2DM patient may suffer from losing weight, fatigue, excessive sweating, dry mouth, insomnia, red tongue, thin coating, and vacuous and rapid pulse and may be differentiated with the syndrome of dual deficiency of qi and yin. The condition was specific to the individual and appropriate treatment was suggested. Syndrome differentiation is the most remarkable characteristic in TCM, and all diagnostic and therapeutic methods of TCM are derived from this principle.

However, syndrome differentiation has several limitations. It regulates the patient's physical condition with a holistic approach to health, but the need to relieve the patient's most painful symptoms is not met in the short term. Moreover, several diseases are found before the appearance of signs and symptoms, which leads to “no syndrome may differentiate.” According to these reasons, more attention should be paid to alleviate the main symptoms. A symptom is a characteristic sign of a particular disease and is a (bodily or mental) phenomenon, circumstance, or change in condition arising from and accompanying a disease or another pathological condition [14], which includes the subjective perception of patients, as well as objective indicators of diseases obtained from testing methods. In ancient China, physicians treated diseases mainly by directly improving symptoms. Some herbal classics described herb efficacies by alleviating the main symptoms; for example, Chuanwu (*Radix Aconiti Praeparata*) may alleviate pain, Banxia (*Rhizoma Pinelliae*) may alleviate nausea and vomiting, Walengzi (*Concha Arcae*) may relieve gastric hyperacidity, and so on. There are several advantages in aiming at main symptoms. First, it is an effective way to relieve the most painful symptoms directly. For example, some diabetic patients also have erectile dysfunction (ED), which may be the most painful symptoms to male patients. Chuanxiong (*Rhizoma Chuanxiong*) and Wugong (*Scolopendra*) were first considered to improve this symptom directly, and subsequently other formulas and herbs were added to constitute a complete prescription. Secondly, difficult diseases always have a complicated etiology and pathogenesis, which results in difficulties with syndrome differentiation; “treating aimed at main symptoms” has the advantage of simplifying the differentiated process and reversing the trends of acute disease directly, thus achieving great clinical efficacy. Thirdly, it could solve the problem of “no syndrome may differentiate”; patients who did not show obvious symptoms in the clinic were found to have abnormal blood lipid indicators and can be treated with herbs such as Shanzha (*Fructus Crataegi*), Hongqu (*Red konjac powder*), and Wuguchong (*Oriental latrine fly larvina*) aimed at hyperlipidemia.

As mentioned above, the characteristics of a syndrome are relatively widespread and abstract, which is easy to conceal the difference [9–11]. The following example may help to explain the shortage of syndrome differentiation. Tuberculosis, lung cancer, diabetes, and chronic nephritis all have a similar syndrome of dual deficiency of qi and yin, but the pathogenesis and prognosis of the above diseases are different, thereby indicating that they should be treated with the same TCM method of boosting qi and nourishing yin; however, this treatment may have insufficient effects on the diseases. This is why syndrome differentiation has strong effects on improving the syndrome but poor effects on treating diseases. Thus, more attention has to be paid to treating diseases. A disease is a condition of poor (more or less seriously) health [9–11]. Disease differentiation provides the main direction for treatment and improves the specificity of the treatment. A lot of the attention should be paid to pathological characteristics of the disease as well as objective indicators such as X-rays and ultra sound. For example, when diabetes, lung cancer, and tuberculosis were found to have similar syndrome of dual deficiency of qi and yin, based on the principle of boosting qi and nourishing yin, the treatment of diabetes may lower the blood glucose, while the treatment for lung cancer may help fight the tumor, and the treatment in tuberculosis is for eliminating* M. tuberculosis*.

**Figure 3:**
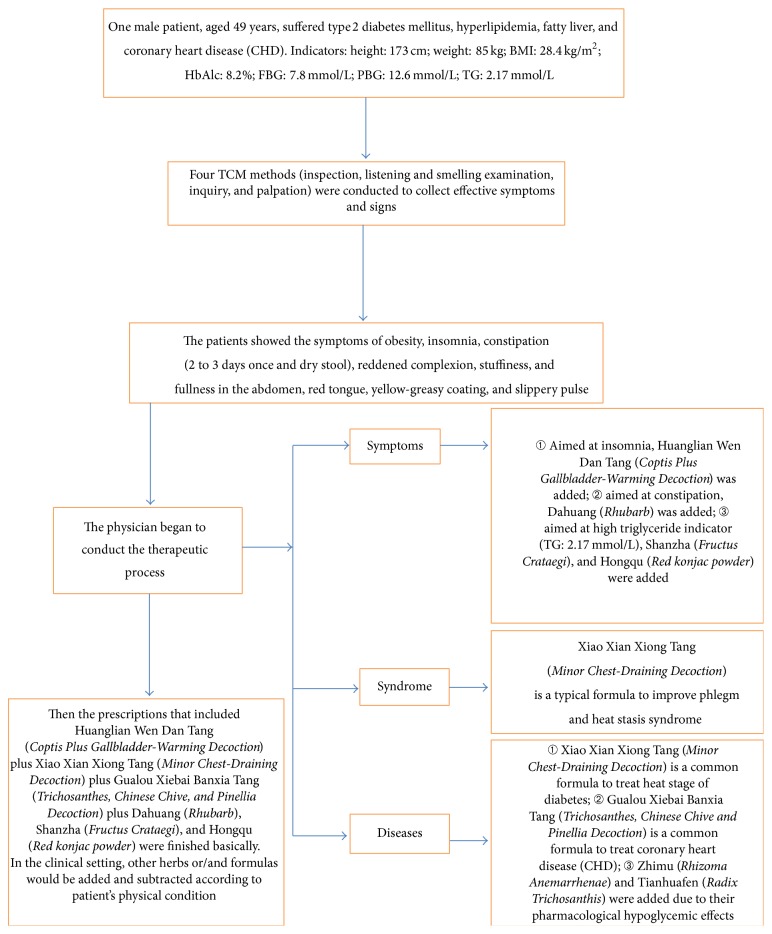
The clinical application of the new mode.

Currently, the combination of symptoms, syndromes, and diseases has become a common mode in the diagnosis and treatment of TCM [9–11], which focuses on “treating aimed at main symptoms directly,” “highlighting chemical or biochemical indicators,” “choosing the formulas that treat both TCM syndromes and diseases in Western medicine,” “emphasizing etiology, pathogenesis, and diagnosis of diseases in Western medicine,” and so on. It is an important way for TCM merging with modern clinical treatment. The following example may help to understand the mode of combination of symptoms, syndromes, and diseases, and the details are in Figure 3.

## 3. Method: Two Important and Innovative Therapeutic Methods

### 3.1. Differences Exist between Modern Clinical Features of T2DM and the “Three Excess and One Loss” of Traditional Xiaoke Disease

In traditional Chinese medicine (TCM), DM may fall under the categories of “Xiaoke disease” and others. It is characterized by excessive drinking, excessive food consumption, excessive urination, and weight loss. All of these symptoms are commonly referred to as “three excess and one loss.” The main pathogenesis lies in yin deficiency leading to endogenous dryness-heat in the body, and blood stasis and phlegm retention are often present. If prolonged yin deficiency impairs yang, dual deficiency of qi and yin as well as dual deficiency of yin and yang will occur. Therefore, the main TCM therapeutic methods for Xiaoke disease are invigorating qi, nourishing yin, clearing away the heat, and promoting fluid production [3, 15, 16]. Famous formulas including Yuye Tang, Bai Hu Jia Renshen Tang, and Jin Gui Shen Qi Wan are widely used [3, 4, 15, 16]. In recent years, several studies have also demonstrated that the distinctive symptoms of T2DM are the “three excess and one loss” [17]. However, modern clinics have found several new features of patients with T2DM, which are as follows. First, 50% of patients with T2DM are without any symptoms, while the diabetic symptoms are not typical in 80% of patients [18]. Clark et al. [19] showed that patients who controlled the blood glucose poorly presented with diabetic symptoms that are defined by the American Diabetes Association (ADA), while patients who controlled their blood glucose well during the early stage of T2DM had no symptoms. Su and Yang [20] proposed that the symptoms of “three excess and one loss” were only manifested in patients with moderate to severe degrees of T2DM. In ancient times, the diagnosis of Xiaoke disease was primarily based on the symptoms of patients, Xiaoke disease could only be diagnosed when these distinctive symptoms appeared, and there was no intervention with Western hypoglycemic drugs. Nowadays, it is often physical examination that leads to the diagnosis of T2DM before the appearance of the “three excess and one loss,” even in prediabetes. The examination of blood glucose is convenient and easy. The early interventions by Western hypoglycemic drugs are common, and the therapeutic methods achieve continuous optimization. Secondly, overweight or obese patients are the main population that suffers from T2DM. In European and American countries, approximately 85% of T2DM patients are overweight or obese, and only 15% of them are normal or thin [21], which is similar to the situation in China. Sixty percent of patients with T2DM also have dyslipidemia. Hyperglycemia, hyperlipidemia, obesity, and fatty liver always occur in combination and cause diseases. Thirdly, patients with the sthenia syndrome outnumber the cases with asthenia syndromes, and internal heat is the core pathogenesis of obese T2DM in the early and middle stages of T2DM [8, 22, 23]. Due to the changes in the etiology, pathogenesis, diagnosis, and interventions between traditional Xiaoke disease and T2DM, new therapeutic methods have been proposed to adapt to the clinical need.

### 3.2. Emphasizing Heat-Clearing in the Early and Middle Stages of T2DM

According to the above, the basic pathogenesis in the early and middle stages of T2DM is associated with “heat”; some scholars have also proposed a concept of “toxin,” such as “glucose toxin” (too much sugar), “lipid toxin” (too much fat), or too many “inflammatory actors,” which refers to the excessive harmful substances in the body of type 2 diabetic patients due to overintake of sweet and greasy food [24, 25]; therefore, “heat” and “toxin” are considered important factors leading to DM. Huang Lian (*Rhizoma Coptidis*) is the classical heat-clearing and detoxifying herb for DM, and berberine (BBR) is an important active component of Huang Lian [26]. Yin et al. [27] investigated the clinical efficacy and safety of BBR in a pilot study. Thirty-six adults with newly diagnosed T2DM were randomly assigned to BBR or metformin treatment (500 mg three times a day) in a 3-month study. Results showed that BBR significantly lowered hemoglobin A1c (HbA1C), fasting blood glucose (FBG), postload plasma glucose (PBG), and TG in patients with T2DM (*P* < 0.05 or *P* < 0.01). Forty-eight adults with poorly controlled T2DM were treated with supplemental BBR for 3 months in a second study. There was a significant decrease in the level of blood glucose and lipids, indicating that the hypoglycemic effect of BBR was similar to that of metformin. Green tea has the property of cold and lowers the fire [28], and some studies have provided evidence that drinking tea could improve insulin resistance and ameliorate the potential risk for T2DM [29, 30]. Current medicine has generally accepted that DM is usually associated with chronic subclinical inflammation [31]. The role of inflammation in the pathogenesis of T2DM and its vascular complications has been confirmed by several studies [32]. Traditional Chinese herbs and formulas usually exert the hypoglycemic effects by controlling inflammation; some heat-clearing and detoxifying herbs and formulas especially possess anti-inflammatory effects. Many studies have shown that heat-clearing herbs could control the blood glucose by inhibiting inflammation, such as Huang Lian (*Rhizoma Coptidis*), GeGen (*Radix Puerariae*), ZhiMu (*Rhizoma Anemarrhenae*), and Tian Hua Fen (*Radix Trichosanthis*) [4, 32]. Huang-Lian-Jie-Du-Tang (HLJDT) is the classical heat-clearing and detoxifying formula used for diabetes [33]. Current medicine has shown that it exhibits anti-inflammatory effects in BALB/c mice and carrageenan-induced mice by inhibiting the production or expression of malondialdehyde (MDA), superoxide dismutase (SOD), nitric oxide (NO), prostaglandin E (2) (PGE2), tumor necrosis factor-*α* (TNF-*α*), and interleukin-6 (IL-6) to lower the blood glucose [34–37]. The method of “Kaiyu Qingre (dissipate stagnation of qi and clear away the heat)” has been proposed according to evidence-based medicine; in one research on observing the Chinese herbal medicine on obese type 2 diabetic patients, Kaiyu Qingre Jiangzhuo formula (KQJF) was given to the treatment group, while metformin was given to the control group. The results showed that there was no significant difference statistically between two groups on lowering the blood glucose (*P* > 0.05) [38]. It is the first evidence of Chinese herbal medicine on lowering the blood glucose in the clinic [8].

### 3.3. Invigorating Blood Circulation throughout the Entire T2DM Process

There are different degrees of vascular lesions in 50% of patients with newly diagnosed T2DM, although some patients' symptoms are atypical [39]. T2DM subclinical vascular lesions are caused by abnormal glycolipid metabolism, oxidative stress, inflammatory factors, insulin resistance, and so on, which exist throughout the entire T2DM process and gradually lead to diabetic complications. Complicated lesions may involve many organs, such as heart, brain, kidneys, retina, nervous system, and skin [40]. Vascular lesions may be considered collateral damage in TCM; the pathogenesis of collateral damage changes from collateral qi stagnation to collateral blood stasis, then to collateral blockage, and finally to collateral damage [41–43]. Sublingual collateral vessels are generally observed to determine the degree of collateral damage, and the observation includes the following two aspects: the body and the color of collateral vessels [42]. Treatment should be aimed at improving the blood circulation and removing obstruction in vessels [41, 42]. Luotong (modified Di Dang Tang, mainly composed of Dahuang (*Rhubarb*), Shuizhi (*Hirudo*), and Taoren (*Semen Persicae*)) is widely applied to activate blood and unblock the collaterals. In the clinical setting, combined therapies of hypoglycemia and Luotong could slow down the progression from impaired glucose tolerance (IGT) to diabetes [42]. Tang-Luo-Ning (TLN, mainly composed of Huangqi (*Radix Astragali*), Danshen (*Radix et Rhizoma Salviae Miltiorrhizae*), and Chishao (*Radix Paeoniae Rubra*)) is used to activate blood and unblock the collaterals. Animal experiments have shown that treatment with TLN may be helpful in delaying the progression of diabetic peripheral neuropathy (DPN) by exerting a neural protection effect [44]. Tong Xin Luo (TXL) has been used in patients with diabetic nephropathy (DN) and has been registered in the State Food and Drug Administration of China. TXL showed positive effects on decreasing the 24-hour urine albumin excretion ratio (24 h UAER) and blood urea nitrogen (BUN). In the treatment of early DN, TXL could improve renal microcirculation, reduce Cys-C and UAER, and delay the progression of renal damage. The mechanism may be related to inhibition of TGF-*β*1-induced epithelial-to-mesenchymal transition in DN [45]. A type of aqueous extract of Huangqi (*Radix Astragali*), Danggui (*Angelica sinensis*), and Sanqi (*Panax notoginseng*) with the therapeutic efficacy of nourishing the blood and invigorating the blood is effective in preventing diabetic retinopathy (DR) by inhibiting leukocyte adherent to the vascular wall, attenuated vascular leakage, and formation of acellular capillaries [46].

## 4. Formula and Herbs: The Combination of TCM Theory and Current Medicine

### 4.1. Treatment Based on Syndrome Differentiation

The first guideline for DM was published in 2007, named* Guideline of Prevention and Treatment of Diabetes by TCM*. With the unification of TCM terminology, diabetes-related terminology became gradually normalized and standardized [8]. Generally speaking, the stagnation stage that represents the body is in a state of congestion and stagnation during the early period of DM, which could be differentiated into syndromes of qi stagnation due to liver depression and spleen and stomach congestion. Xiaoyao Powder or Houpo Sanwu Tang is recommended to dissipate the stagnation and remove the congestion. The heat stage represents the development of diseases and could be seen more as the sthenia syndrome during the early or middle periods of T2DM. Based on the comprehensive and systemic review, peer review, validation sessions, and analysis of literature, the TCM clinical guidelines were finally formulated, and the heat stage was differentiated into six common clinical syndromes. Heat-clearing and fire-draining are important therapeutic methods. Da Chaihu Tang is commonly recommended for liver and stomach stagnated heat syndromes, Bai Hu Tang for lung and stomach exuberant heat syndromes, Dahuang Huanglian Xie Xin Tang for stomach and intestine excessive heat syndromes, Gegen Qin Lian Tang for intestinal damp and heat syndromes, Xiao Xian Xiong Tang for phlegm and heat stasis syndromes, and San Huang Tang plus Wu Wei Xiao Du Yin for intense heat toxin syndrome. The deficiency stage represents the further development of the disease and could be seen in more syndromes of asthenia and sthenia in complex middle or late periods of diabetes. The deficiency stage can be differentiated into five syndromes, including deficiency of body liquid due to excessive heat, effulgent fire due to yin deficiency, dual deficiency of qi and yin, spleen deficiency and stomach congestion, and cold and heat in complexity. The key points of treatment in this stage are supplementing the deficiency and eliminating the excess. Bai Hu Jia Renshen Tang, Zhi Bai Dihuang Wan, Sheng Mai Yin plus Zeng Ye Tang, Banxia Xie Xin Tang, or Wumei Wan is recommended. The damage stage represents the end of the disease. At this stage, the functions of the* zang-fu* organs become gradually weaker, and some pathological factors accumulate, such as phlegm, turbid, stasis, or toxin. The treatment should be based on regulating* yin *and* yang*. The damage stage could be differentiated into liver-kidney yin deficiency, dual deficiency of yin and yang, and spleen-kidney yang deficiency syndromes. Qi Ju Dihuang Wan, Jin Gui Shen Qi Wan, and Fuzi Li Zhong Wan are commonly used [22]. The details are shown in Table 1.

### 4.2. Choosing Herbs of Bitter and Sour Flavors to Counteract Sweet Flavor

According to TCM theory, four-qi and five-flavor theory are one of the basic concepts, and the method of “bitter and sour flavors to counteract sweet flavor” is a great approach to lower blood glucose levels. Bitter flavor is in direct opposition to sweet flavor, and sour flavor can neutralize sweet flavor [47]. Herbs with bitter and sour flavors are excellent when used to treat hyperglycemia. Herbs with a bitter flavor are based on San Huang Tang, Longdancao (*Radix et Rhizoma Gentianae*), Kushen (*Radix Sophorae Flavescentis*), Kuding (*Herba Corydalis Bungeanae*), and Shanzhizi (*Fructus Gardeniae*) and also could be considered, which generally include bitter flavor and cold property. Herbs of sour flavor are represented by Fructus Mume formula, Shanzhuyu (*Fructus Corni*), Suanzaoren (*Semen Ziziphi Spinosae*), and Shiliupi (*Pericarpium Granati*) and should also be considered [48].

### 4.3. Choosing Formulas and Herbs Aimed at Main Symptoms

Alleviating the main symptoms is important in treating T2DM and its complications; thus, the selection of formulas and herbs should be based on the main symptoms. For example, vomiting is the most troublesome problem for diabetics with severe gastroparesis (DGP), Xiao-Banxia-Tang combined with Suye Huanglian Yin is commonly used to relieve nausea and vomiting [49–51]. Proteinuria and edema are obvious symptoms for DN, which could be improved by Liuwei Dihuang Decoction [52, 53] and large amounts of Huangqi (*Radix Astragali*), Danshen (*Salvia miltiorrhiza*), and Fuling (*Poria*) [54, 55]. DPN patients with acral numbness and pain could be treated with Huangqi Guizhi Wu Wu Decoction [56] and large amounts of Chuanwu (*Radix Aconiti Praeparata*) [57] to improve symptoms and increase nerve conduction velocities (NCVs). The details showed in Table 3.

### 4.4. Applying Modern Pharmacological Achievements

With the development of modern pharmacological products, the effective components provide evidence for herbs or formulas to treat diseases [58]. There are several herbs that possess definite hypoglycemic effects and are often used in the traditional Chinese formulas for T2DM and its complications, including Huanglian (*Rhizoma Coptidis*), Huangqin (*Radix Scutellariae*), Renshen (*Radix et Rhizoma Ginseng*), Zhimu (*Rhizoma Anemarrhenae*), and Tianhuafen (*Radix Trichosanthis*) [4, 16]. Details are shown in Table 2. Some herbs have great effects on improving other indicators, Weilingxian (*Radix et Rhizoma Clematidis*) may lower the blood uric acid [59], Wuweizi (*Fructus Schisandrae Chinensis*) may lower the aminotransferase [60], and Yinchen (*Herba Artemisiae Scopariae*) and Huzhang (*Rhizoma Polygoni Cuspidati*) may improve fatty liver [61, 62]. In the clinical setting, the application of pharmacological products plays an important role in the treatment of DM.

## 5. Importance of Drug Dose in the Treatment of Diabetes

The therapeutic efficacy of TCM may be not only determined by syndrome differentiation, formula compatibility, medicinal properties and quality, water decoction, and administration method but also closely related to the applicable drug dose. As the saying goes, “the secret of traditional Chinese medicine is in the dose,” the dose of herbs has always been difficult to study [63].

According to traditional concept, Chinese herbal medicines are only considered supplementary treatment for lowering the blood glucose. However, we have confirmed that Chinese herbal medicine possesses independent antihyperglycemic effects based on large scales of randomized controlled trials (RCTs), and adverse events were less common than with metformin [38]. The key point to lowering the blood glucose independently is dose. In our previous study, we demonstrated the relationship between dose and effect through RCTs. One hundred and eighty-seven T2DM patients were randomly allocated to receive high (HD, *n* = 44), moderate (MD, *n* = 52), and low doses (LD, *n* = 50) of Gegen Qin Lian Decoction or the placebo (*n* = 41) for 12 weeks. Patients that received the HD or MD showed significant difference in adjusted mean changes from baseline of HbA1c and FBG compared with the LD and placebo groups. The dose-effect relationship is obvious [64]. Huanglian is commonly used in the heat and deficiency stages of T2DM [22]. Liu made a survey of the dose of Huanglian in 1,321 effective formulas (when the decreased percentage of FBG and PBG was >20% of those before treatment or the decreased percentage of HbAlc was >10% of that before treatment within 12 weeks, the formula was thought of as an effective formula, and other else was thought of as an ineffective formula) to treat T2DM, and the result showed that commonly recommended dose of Huanglianwas 15 g when FBG < 7 mmol/L, 30 g when FBG < 10 mmol/L, and 30 g to 45 g when FBG was ≥10 mmol/L [48]. There is a positive correlation between the dose of Huanglian and the decrease of blood glucose.

Chuanwu (*Radix Aconiti Praeparata*) is commonly used in the treatment of DPN with severe acral pain, tingling, and cold. The recommended dose of Chuanwu (*Radix Aconiti Praeparata*) should be “15–60 g,” even to a maximum dose of 120 g for alleviating the pain, whereas the routine dose of “1.5–3 g” in Chinese Pharmacopoeia (2010 edition) is usually ineffective. The decocted time of Chuanwu (*Radix Aconiti Praeparata*) should be more than 60 mins, and medicinal compatibility with Gancao (*Radix et Rhizoma Glycyrrhizae*) or Baimi (*Mel*) is also necessary to resolve toxins [57]. Banxia (*Rhizoma Pinelliae*) and Shengjiang (*Rhizoma Zingiberis Recens*) are often used for treating DGP nausea and vomiting. The routine doses of Banxia (*Rhizoma Pinelliae*) and Shengjiang (*Rhizoma Zingiberis Recens*) are “3–9 g” and “3–9 g,” respectively, whereas the recommended dose of Banxia (*Rhizoma Pinelliae*) should be “15–60 g,” and the dose of Shengjiang (*Rhizoma Zingiberis Recens*) should be “15–30 g” [50, 65, 66].

## 6. Discussion

With the increasing incidence of obesity, T2DM is likely to become even more prevalent in the future. It has a significant impact on the quality of life and the number of deaths as well as on the financial resources of the public health care system. Currently, CAM therapies are widespread in both developing and developed countries. Due to positive views of patients regarding CAM therapies and the increased availability of them, they are frequently used for T2DM globally [67]. The commonly used CAM therapies include Chinese herbal medicines, acupuncture, nutritional supplements and advice, spiritual healing, and relaxation techniques [7]. Recently, treating obese T2DM with acupuncture has become popular, and a lot of progress has been made to indicate that acupuncture is safe and effective [68]. Chinese herbal medicine contains various active ingredients, which could provide multiple therapeutic effects on multiple targets, such as enhancement of insulin sensitivity, stimulation of insulin secretion, or reduction of carbohydrate absorption [16]. Chinese herbal medicines could also help treat the diabetic complications by ameliorating abnormalities related to blood viscosity, microcirculation, and oxidative stress [69]. In the light of recent studies, it is not difficult to find that the etiology, pathogenesis, and therapeutic strategies of diabetes have been changed recently. With the development of modern diagnosis and treatment on DM, the thoughts of highlighting the combination of symptoms, syndromes, and diseases, reunderstanding the etiology and pathogenesis of diabetes, emphasizing heat-clearing and invigorating blood circulation, and choosing formula and herbs are based on the combination of TCM theory and current medicine, and paying attention to dosage has been gradually and widely accepted; only by adopting these thoughts, the clinical efficacy of Chinese formulas and herbs on DM may be improved. We have confirmed the effects of formulas and herbs on regulating metabolic problems from integrated perspectives. For example, obese diabetes patients have hyperglycemia along with fatty liver, hyperlipidemia, hypertension, hyperuricemia, and other metabolic disorders. Western medicine has not found an effective way to treat the metabolic syndrome; each abnormality has been treated separately. Here, we take advantage of TCM with a holistic approach. Furthermore, formulas and herbs may also reverse risk factors leading to diabetes. In one study, we observed that the Chinese herbal formula Tianqi Jiang Tang Capsule reduced progression from impaired glucose tolerance (IGT) to diabetes. After a 12-month treatment, results demonstrated that Tianqi significantly decreased the incidence of T2DM in subjects with IGT by 32.1% compared with placebo [70]. There are also diverse Chinese patent drugs commonly used for treating DM clinically, including Xiaoke Wan, Jiangtangjia Pian, YuquanWan, and Tangmaikang Keli, which also play an important role [4]. In the clinical setting, a large amount of clinical experience has been accumulated, and these innovative thoughts have been gradually accepted and promoted the development of TCM. The emergence of evidence-based medicine (EBM) has provided objective efficacy assessment of TCM with new thoughts and methods [71]; well-designed, large-scale, high-quality multicenter RCTs are still required to provide stronger evidence in the future. With continuous efforts, TCM will undoubtedly play a more important role in fighting T2DM.

## Figures and Tables

**Table 1:** Classical formulas and Chinese herbs recommended for T2DM treatment.

Stage	Syndrome	Formula	Efficacy	Components
Stagnation	Qi stagnation due to liver depression	Xiaoyao Powder	Soothing the liver, dissipating stagnation of qi	*Radix Bupleuri*, *Radix Angelicae Sinensis*, *Yam*, *Atractylode*s, *Poria cocos*,* Glycyrrhiza*,* Herba Menthae*, *Rhizoma Zingiberis Recens. *
	Spleen and stomach congestion	Houpo Sanwu Tang	Moving qi, removing food stagnation	*Officinal Magnolia Bark*, *Rhubarb*, *Gardenia. *

Heat	Liver and stomach stagnated heat	Da Chaihu Tang	Clearing liver heat, draining stomach fire	*Radix Bupleuri*, *Radix Scutellariae*, *Rhubarb*,* Gardenia, Yam*, *Rhizoma Pinelliae*, *Rhizoma Zingiberis Recens*, *Fructus Jujubae. *
	Lung and stomach exuberant heat	Bai Hu Tang	Clearing lung heat, engendering fluids to quench thirst	*Gypsum Fibrosum*, *Rhizoma Anemarrhenae*, *Oryza sativa *L., *Glycyrrhiza. *
	Stomach and intestine excessive heat	Dahuang Huanglian Xie Xin Tang	Draining stomach and intestine fire	*Rhubarb*, *Rhizoma Coptidis*, *Radix Scutellariae. *
	Intestinal damp and heat	Gegen Qin Lian Tang	Clearing heat and draining dampness	*Pueraria*, *Rhizoma Coptidis*, *Radix Scutellariae*,* Glycyrrhiza. *
	Phlegm and heat stasis	Xiao Xian Xiong Tang	Clearing heat and dissolving phlegm	*Rhizoma Coptidis*, *Rhizoma Pinelliae*, *Semen Trichosanthis. *
	Intense heat toxin	San Huang Tang plus Wu Wei Xiao Du Yin	Draining fire and resolving toxins	*Rhizoma Coptidis*, *Radix Scutellariae*, *Rhubarb*, *Flos Lonicerae Japonicae*, *Flos Chrysanthemi Indici*, *Herba Taraxaci*, *Herba Violae*, *Herba Begoniae Fimbristipulatae. *

Deficiency	Deficiency of body liquid due to excessive heat	Bai Hu plus Renshen Tang	Clearing lung heat, promoting fluid production	*Gypsum Fibrosum*, *Rhizoma Anemarrhenae*, *Oryza Sativa L.*, *Glycyrrhiza*, *Ginseng. *
	Effulgent fire due to yin deficiency	Zhi Bai Dihuang Wan	Enriching yin, clearing the fire	*Rhizoma Anemarrhenae*, *Cortex Phellodendri*, *Radix Rehmanniae*, *Radix Asparragi Officinalis*, *Rhizoma Dioscoreae*, *Poria cocos*, *Rhizoma Alismatis*, *Cortex moutan*.
	Dual deficiency of qi and yin	Sheng Mai Yin plus Zeng Ye Tang	Boosting qi and nourishing yin	*Ginseng*, *Radix Ophiopogonis*, *Fructus Schisandrae Chinensis*, *Radix scrophulariae*, *Radix Rehmanniae*.
	Spleen deficiency and stomach congestion	Banxia Xie Xin Tang	Dispersing stagnation with bitter-acrid medicinals	*Rhizoma Pinelliae*, *Zingiberis*,* Ginseng*, *Rhizoma Coptidis*, *Radix Scutellariae*,* Fructus Jujubae*, *Glycyrrhiza*.
	Cold and heat in complexity	Wumei Wan	Clearing the upper and warming the lower	*Fructus Mume*, *Herba Asari*, *Zingiberis*, *Rhizoma Coptidis*, *Radix Angelicae Sinensis*, *Typhonii Gigantei*, *Fructus Zanthoxyli*, *Ramulus Cinnamomi*, *Ginseng*, *Cortex Phellodendri*.

Damage	Liver-kidney yin deficiency	Qi Ju Dihuang Wan	Enriching and nourishing the liver and kidney	*Fructus Lycii*, *Flos Chrysanthemi*, *Radix Rehmanniae*, *Radix Asparragi Officinalis*, *Rhizoma Dioscoreae*, *Poria cocos*, *Rhizoma Alismatis*,* Cortex moutan*.
	Dual deficiency of yin and yang	Jin Gui Shen Qi Wan	Enriching yin and supplementing yang	*Typhonii Gigantei*, *Cortex Cinnamomi*, *Radix Rehmanniae*, *Radix Asparragi Officinalis*, *Rhizoma Dioscoreae*, *Poria cocos*, *Alisma*, *Cortex moutan*.
	Spleen-kidney yang deficiency	Fuzi Li Zhong Wan	Warming and supplementing the spleen and kidney	*Typhonii Gigantei*, *Zingiberis*, *Ginseng*, *Atractylodes*, *Glycyrrhiza. *

**Table 2:** Classifications of function of herbal medicines possessing hypoglycemic efficacy.

TCM efficacies	Herbal medicines
Clearing heat	Huanglian (*Rhizoma Coptidis*), Tianhuafen (*Radix trichosanthis*), Zhimu (*Rhizoma Anemarrhenae*), Huangbai (*Cortex Phellodendri*), Gegen (*Radix Puerariae*), Kugua (*Fructus Balsampear*), Shigao (*Gypsum Fibrosum*), Huangqin (*Radix Scutellariae*), Zhizi (*Fructus Coini*), Digupi (*Cortex Lycii Radicis*), Lugen (*Rhizoma Phragmitis*)

Nourishing yin (promoting body fluids production)	Dihuang (*Radix Rehmanniae*), Shanzhuyu (*Radix Asparragi Officinalis*), Wumei (*Fructus Mume*), Yuzhu (*Rhizoma Polygonati Odorati*), Maidong (*Radix Ophiopogonis*), Gouqizi (*Fructus Lycii*), Nvzhenzi (*Fructus Ligustri Lucidi*), Wuweizi (*Fructus Schisandrae*), Shihu (*Herba Dendrobii*), Shengmuli (*Concha Ostreae*), Xuanshen (*Radix Scrophulariae*)

Invigorating qi (fortifying the spleen)	Huangqi (*Radix Astragali seu Hedysari*), Renshen (*Radix Ginseng*), Huangjing (*Rhizoma polygonati*), Cangzhu (*Rhizoma Atractylodis*), Shanyao (*Rhizoma Dioscoreae*), Yiyiren (*Semen Coicis*)

Activating stasis	Danshen (*Radix Salviae Miltiorrhizae*), Sanqi (*Radix Notoginseng*), Guijianyu (*Ramulus Euonymi*), Chishao (*Radix Paeoniae Rubra*), Shuizhi (*Hirudo*), Chuanxiong (*Rhizoma Ligustici Chuanxiong*), Danggui (*Radix Angelicae Sinensis*), Taoren (*Semen Persicae*)

Warming yang	Tusizi (*Semen Cuscutae*), Yinyanghuo (*Herba Epimedii*), Dongchongxiacao (*Cordyceps*), Bajitian (*Radix Morindae oficinalis*), Roucongrong (*Herba Cistanches*), Dasuan (*Allii Sativi Bulbus*), Buguzhi (*Fructus Psoraleae*), Fuzi (*Radix Aconiti Lateralis Praeparata*)

Draining water	Zexie (*Rhizoma Alismatis*), Fuling (*Poria cocos*), Yumixu (*Stigma Maydis*), Dongguapi (*Exocarpium Benincasae*)

**Table 3:** Chinese herbal formulas mentioned in the review.

Formulas	Components
Yuye Tang	Shanyao (*Rhizoma Dioscoreae*), Huangqi (*Radix Astragali seu Hedysari*), Zhimu (*Rhizoma Anemarrhenae*), Jineijin (*Endothelium Corneum Gigeriae Galli*), Gegen (*Radix Puerariae*), Wumei (*Fructus Mume*), Tianhuafen (*Radix Trichosanthis*)

Xiao Banxia Tang	Banxia (*Rhizoma Pinelliae*), Shengjiang (*Rhizoma Zingiberis Recens*)

Suye Huanglian Yin	Huanglian (*Rhizoma Coptidis*), Zisuye (*Folium Perillae*)

Huangqi Guizhi Wu Wu Tang	Huang Qi (*Radix Astragali seu Hedysari*), Gui Zhi (*Ramulus Cinnamomi*), Shanyao (*Rhizoma Dioscoreae*), Sheng Jiang (*Rhizoma Zingiberis Recens*), Da Zao (*Fructus Jujubae*)

Di Dang Tang	Dahuang (*Rhubarb*), Shuizhi (*Hirudo*), Taoren (*Semen Persicae*), Mangchong (*Tabanus*)

## Abbreviations

DM: Diabetes mellitus
T2DM: Type 2 diabetes mellitus
TCM: Traditional Chinese medicine
BBR: Berberine
HbAlC: Hemoglobin A1c
FBG: Fasting blood glucose
PBG: Postprandial blood glucose
CHO: Cholesterol
TG: Triglyceride
HDL-C: High-density lipoprotein
LDL-C: Low-density lipoprotein
BMI: Body mass index
Cr: Creatinine
BUN: Blood urea nitrogen
RCTs: Randomized controlled trials.
